# Ketosis-Prone Type 2 Diabetes Mellitus: An Unusual Presentation

**DOI:** 10.7759/cureus.30031

**Published:** 2022-10-07

**Authors:** Lana Makahleh, Ahmad Othman, Venkata Vedantam, Neethu Vedantam

**Affiliations:** 1 Internal Medicine, East Tennessee State University Quillen College of Medicine, Johnson City, USA; 2 Infectious Diseases, East Tennessee State University Quillen College of Medicine, Johnson City, USA

**Keywords:** ketosis-prone diabetes, type 2 diabetes, diabetic ketoacidosis, ketosis, ketosis-prone type 2 diabetes, diabetes

## Abstract

Ketosis-prone type 2 diabetes is a form of diabetes that usually presents with diabetic ketoacidosis (DKA) in patients who are not insulin dependent. It is commonly seen in African, African American, and Hispanic populations. Although the pathogenesis is not fully understood yet, it is believed to be caused by stress-induced reversible beta-cell and alpha-cell dysfunction in the pancreas. Here, we describe the case of an 80-year-old white female with well-controlled type 2 diabetes mellitus who had unexplained DKA in the setting of a urinary tract infection. The patient’s DKA resolved after administering appropriate therapy, and she did not require treatment with insulin on discharge.

## Introduction

Diabetic ketoacidosis (DKA) is a well-known complication of type 1 diabetes mellitus (T1DM) [1]. It is also seen in some insulin-dependent patients with advanced type 2 diabetes mellitus (T2DM) [1]. However, a subset of patients with T2DM develops DKA in the face of acute physiologic stress due to reversible failure of β-cell function [2]. Patients with ketosis-prone type 2 diabetes (KPT2D) lack the characteristic β-cell autoimmunity seen in T1DM [3]. In the initial presentation, there are no reliable features to distinguish between KPT2D and other forms of diabetes. It should be suspected in non-white patients presenting with DKA and not known to have T1DM [4]. Patients with KPT2D need insulin only during periods of physiologic stress, which is otherwise managed with diet or oral hypoglycemic agents [5].

## Case presentation

An 80-year-old female nursing home patient with a medical history significant for dementia, diet-controlled T2DM, hypertension, and myelodysplastic syndrome presented to the emergency department (ED) complaining of nausea, vomiting, and severe abdominal pain.

History was provided by her daughter-in-law who reported that the patient’s symptoms started five days prior to her current hospitalization. At the time, she was brought to the ED and underwent a computed tomography scan (CT) of the abdomen, which did not show any acute pathology. Urinalysis showed that the patient had a urinary tract infection (UTI), and she was sent back to the nursing home on empiric trimethoprim/sulfamethoxazole. A few days later, the patient’s symptoms progressed, and she was brought back. Urine culture obtained from the previous ED visit grew vancomycin-resistant *Enterococcus faecalis* (VRE). On the physical examination, vitals were significant for tachycardia. She appeared very weak with dry mucous membranes and had diffuse abdominal tenderness without guarding or rigidity, heart sounds were normal without any murmurs, and the respiratory examination was benign without any wheezes or crackles. Laboratory findings were significant, as shown in Table 1.

**Table 1:** Laboratory findings. HPF: high-power field; CFU: colony-forming unit

Laboratory studies		Patient values	Reference values
Sodium (mmol/L)		144	136–145
Potassium (mmol/L)		3.3	3.5–5.0
Chloride (mmol/L)		107	98–106
Bicarbonate (mmol/L)		17	22–32
Glucose (mg/dL)		271	70–99
Blood urea nitrogen (mg/dL)		21	6–20
Creatinine (mg/dL)		0.81	0.60–1.10
Anion gap (mmol/L)		23	5–15
Lactic acid (mmol/L)		1.3	0.7–2.1
White blood cell count (K/µL)		12,000	3.5–11
Hemoglobin (g/dL)		11.4	11.7–15.0
Mean cell volume (fL)		102.4	78.0–98.0
Platelet count (K/µL)		311	150–400
Urine drug screen		Negative	Negative
Urinalysis	Clarity	Cloudy	Clear
	Ketones	40	None
	White blood cells	>=100	0–4/hpf
	Red blood cells	0-4	0–4/hpf
	Bacteria	+4	Negative
	Leukocyte esterase	Large leukocyte esterase	Negative
	Nitrates	Negative	Negative
Urine culture		>100,000 CFU/mL Vancomycin-eesistant Enterococcus faecium	Negative
Beta-hydroxybutyrate		>2.00	<0.28
Arterial blood gas findings on Room air		Patient values	Reference values
pH		7.21	7.35–7.45
PCO_2_ (mmHg)		2.6	35–45
PO_2_ (mmHg)		103	82–92
HCO_3_ (mmol/L)		8	22–26
Base excess (mmol/L)		30	-2.0–2.0
O_2_ saturation (%)		98.1	96–100

The patient was diagnosed with DKA based on her labs which showed high anion gap metabolic acidosis, serum glucose of 380 mg/dL, and bicarbonate of 10 mmol/L. She was treated with intravenous (IV) fluids, basal insulin, and sliding-scale insulin. IV thiamine was given as the patient was having episodes of vomiting and decreased oral intake. The repeated metabolic panel showed that potassium and phosphorus levels were low, after which the patient was given IV potassium and phosphorus. Imaging studies of the kidneys were done, which ruled out hydronephrosis. She was also started on linezolid to treat the UTI infection. Linezolid was chosen because her old urine culture from the previous ED visit grew VRE. Repeated urine analysis and culture during her current hospitalization also showed the same growth. She was given linezolid for a total of seven days.

The patient showed significant improvement after starting the treatment. She was able to tolerate oral intake. Bicarbonate went back to normal with an anion gap of <10, and she was discharged three days later.

The patient had a similar presentation three months prior to her hospitalization, with severe DKA in the setting of a UTI, which also resolved with IV fluids, insulin, and antibiotics. In both instances, the patient did not have any insulin requirement after treating her DKA and infection. The patient was discharged without oral hypoglycemic agents because of her well-controlled blood glucose, as evidenced by her hemoglobin A1c, which was 5.7%, as measured during the current hospitalization. The patient was diagnosed with KPT2D and was discharged after counseling the family about this rare phenomenon.

## Discussion

KPT2D is an emerging form of diabetes mellitus that usually presents with DKA or unprovoked ketosis in patients who lack the typical phenotypic features of T1DM [3]. This form of diabetes is also referred to in the literature by various names such as Flatbush diabetes, atypical diabetes, idiopathic type 1 diabetes, and KPT2D [6]. Historically, KPT2D is often reported in African, African American, and Hispanic populations [6]. This condition has also been described among young adult Japanese [7]. Patients with KPT2D are middle-aged, obese, and have a family history of T2DM [4]. These patients usually present with disorders in insulin secretion and insulin sensitivity in the setting of a physiological stressor. Treatment of the precipitating cause, which in many cases is an infection, along with initiation of insulin will lead to an improvement in β-cell function and insulin sensitivity. Eventually, patients can discontinue insulin treatment [8].

Most recent metabolic studies have shown that ketosis in KPT2D is because of a significantly slowed ketone oxidation rate, which results in increased concentrations of beta-hydroxybutyrate and its intracellular metabolites [9]. Studies are still underway to determine the inheritance pattern of KPT2D and whether it is polygenic or monogenic. A cohort study of 15 sub-Saharan Africans who had ketosis-prone diabetes in remission and had been insulin-free for a median of six months was conducted to characterize both β- and α-cell functions. The study showed that these patients also had impaired glucagon suppression in response to oral and IV glucose and arginine compared to controls, suggesting an α-cell dysfunction in addition to a β-cell dysfunction in patients with ketosis-prone diabetes [10].

The clinical importance of diagnosing KPT2D is to distinguish it from T1DM, which requires lifelong insulin treatment [11]. Studies have shown that patients with KPT2D can discontinue insulin therapy eventually as their β-cells recover from glucotoxicity [12]. In a study conducted among 106 patients with KPT2D to evaluate predictive factors favoring insulin discontinuation, it was concluded that having new-onset diabetes and higher β-cell functional reserve (measured as C-peptide-to-glucose ratio at six months >11 nmol/mmol × 100) are the best predictors for insulin discontinuation [13].

Data are still lacking about the optimal treatment regimen for KPT2D once discontinuing insulin therapy. However, progressive uncontrolled hyperglycemia is a potent risk factor for ketotic relapses in KPT2D patients [8]. Thus, insulin-sensitizing agents, such as metformin or thiazolidinediones, are usually prescribed to control blood sugar levels. Sulfonylurea, meglitinide, or α-glucosidase inhibitors can also be employed if blood sugar levels are not within the therapeutic target after eight weeks of treatment [3]. A small clinical trial has shown that metformin and sitagliptin are similarly effective in prolonging normoglycemic remission in KPT2D patients [14].

## Conclusions

Although distinguishing between T1DM and KPT2D can be challenging, it is of great clinical significance. Older obese patients with a family history of T2DM presenting with DKA should raise suspicion of KPT2D. Patients with KPT2D rarely need lifelong insulin therapy, and their blood sugar levels can be controlled with oral hypoglycemic agents along with modification of lifestyle with exercise and diet. Avoiding prolonged insulin therapy in KPT2D patients is helpful as it can avoid the negative effects of insulin therapy and patients can have a better quality of life overall.
